# Supplementary material for: Facing the phase problem in Coherent Diffractive Imaging via Memetic Algorithms
Source: Sci Rep. 2017 Feb 9;7:42236. doi: 10.1038/srep42236 (PMC5299835; doi:10.1038/srep42236)
Supplement: Supplementary Information [file srep42236-s1.pdf]

Supplementary Information

Facing the phase problem in Coherent Diffractive Imaging via Memetic Algorithms

Alessandro Colombo[1], Davide Emilio Galli[1], Liberato De Caro[3], Francesco Scattarella[2], and Elvio Carlino[2]

[1] Universitá degli Studi di Milano, via Giovanni Celoria 16, 20133 Milano
[2] Istituto Officina dei Materiali, Consiglio Nazionale delle Ricerche (IOM-TASC-CNR), Strada Statale 14 km 163.5, 34149 Trieste
[3] Istituto di Cristallografia, Consiglio Nazionale delle Ricerche (IC-CNR), via Giovanni Amendola, 122/O, 70126 Bari, Italy

Phase retrieval on real-valued data

A first test regards real-valued two-dimensional (2D) positive functions in the object space. The standard phasing deterministic approach, which has to be intended as MPR with a genetic fraction $G = 0$, will be compared with MPR with a genetic fraction $G = 1$. Data of size 512x512 pixels have been assumed unaffected by any noise and any lack of information.

The *qualitative* comparison between the two different phasing approaches, the deterministic one with $G = 0$ versus the stochastic MPR with $G = 1$, is directly given by the quality of the reconstructed 2D function. The *quantitative* comparison, instead, is based on a *figure of merit* (FOM) obtained via the equation

$$E[\rho] = D[P_{\mathcal{M}}\rho(\mathbf{x}), P_{\mathcal{S}}\rho(\mathbf{x})] = \int d\mathbf{q}\ [\sqrt{I(\mathbf{q})} - |\mathscr{F}[\Pi\rho](\mathbf{q})|\ ]^2 \tag{S-1}$$

as function of the generations, where the lower the value is, the better the quantitative retrieving of the 2D function. Here, $\mathbf{x} = (x, y)$, with x and y the cartesian components of the position vector x with respect to the reference system. Analogously, $\mathbf{q} = (u, v)$, where u and v are the spatial frequencies components with respect to the reference axes. The meaning of the mathematical symbols is the same of the main text. The 2D function depicted in the FOM represents, in terms of error, the best element in $\{\rho_i(\mathbf{x})\}_{i=1\ldots N_p}$, i.e., the quantity

$$\min_{\rho} E[\rho] \quad \text{where} \quad \rho \in \{\rho_i(\mathbf{x})\}_{i=1\ldots N_p} \tag{S-2}$$

Tests on simulated data make possible a numerical evaluation of the error possible not only by comparing the modulus of trial and true solutions in the Fourier space, via Eq.(S-1), but also by means of a comparison of the trial solution with the true solution directly in the object space.

Indeed, for simulated data, it is possible to define a *true error* E_t whose numerical value is obtained via the following equation:

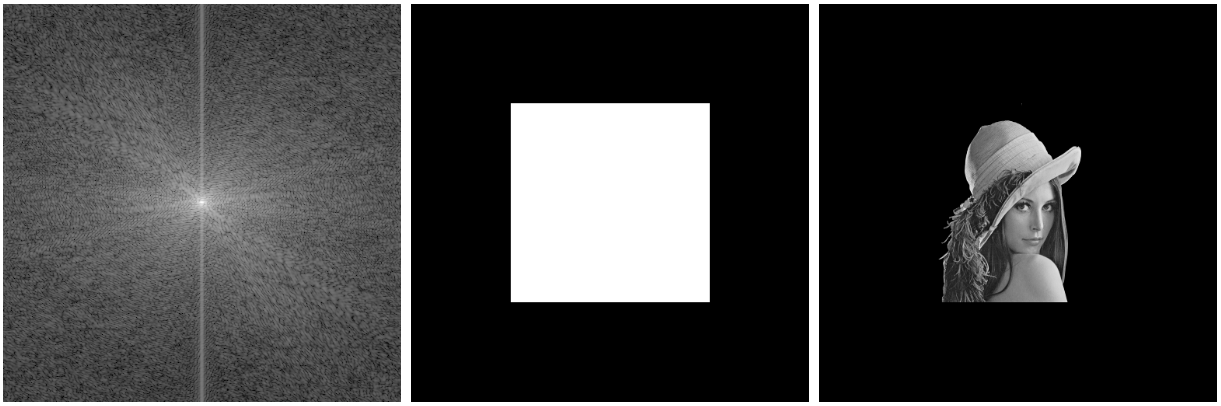

(a) (b) (c)

Figure S-1: Real-valued retrieval. (a) Input diffraction pattern; (b) provided maximum support function S_{max}; (c) true solution.

$$E_t[\rho] = \frac{\int d\mathbf{x}\ |\rho(\mathbf{x}) - \rho_{\mathrm{true}}(\mathbf{x})|}{\int d\mathbf{x}\ |\rho_{\mathrm{true}}(\mathbf{x})|} \tag{S-3}$$

where ρ_{true} and ρ are 2D functions in the object space.

For real-valued 2D positive functions in the object space the algorithm can impose the constraints of *reality* and *positivity*, i.e:

$$\Im[\rho(\mathbf{x})] = 0 \tag{S-4}$$

$$\Re[\rho(\mathbf{x})] = \max[0, \Re[\rho(\mathbf{x})]] \tag{S-5}$$

We will not provide any tight support function S during the phasing process. We will only limit the maximum object space allowed to the trial solution, where it can have non-zero values. This is a loose support function S defined by an area four time smaller than the total area of the direct space, i.e., by a square area of 256x256 pixels. This condition implies a *constraint ratio* $\Omega = 2$, as defined in [S.4]. This area will be denoted as S_{max} and represents a very loose real space constraint. In Fig. S-1 the input diffraction pattern (Fig. S-1a), S_{max} (Fig. S-1b) and the 2D function to be retrieved (Fig. S-1c) are shown.

In particular, the input diffraction pattern is given by the square modulus of the Fourier Transform (FT) of the 2D function to be retrieved (Lena image, adapted from the picture 4.2.04 in the USC–SIPI image database [S.6]). In Fig. S-2 the results of the retrieval process for a small population ($N_p = 512$) are shown: Fig. S-2b is the best output for G=0 (deterministic approach), with a zoom on the panel below; Fig. S-2c is the best output for G=1 (stochastic approach), with a zoom on the panel below; Fig. S-2a is the output of the FOM.

Table S-1 reports the parameters used in this test of MPR which are the same both for G=0 and G=1 to compare the efficiency of the stochastic approach with respect to the deterministic one in the same conditions.

The better result of MPR is not only underlined by the FOM output (Fig. S-2a) but it is also evident from a visual comparison between Fig. S-2b and Fig. S-2c.

To show the influence of the dimension of the population in Fig. S-3 we report on the results obtained for $N_p = 16384$: Fig. S-3b is the best output for G=0 (deterministic approach), with a zoom on the panel below; Fig. S-3c is the best output for G=1 (stochastic approach), with a

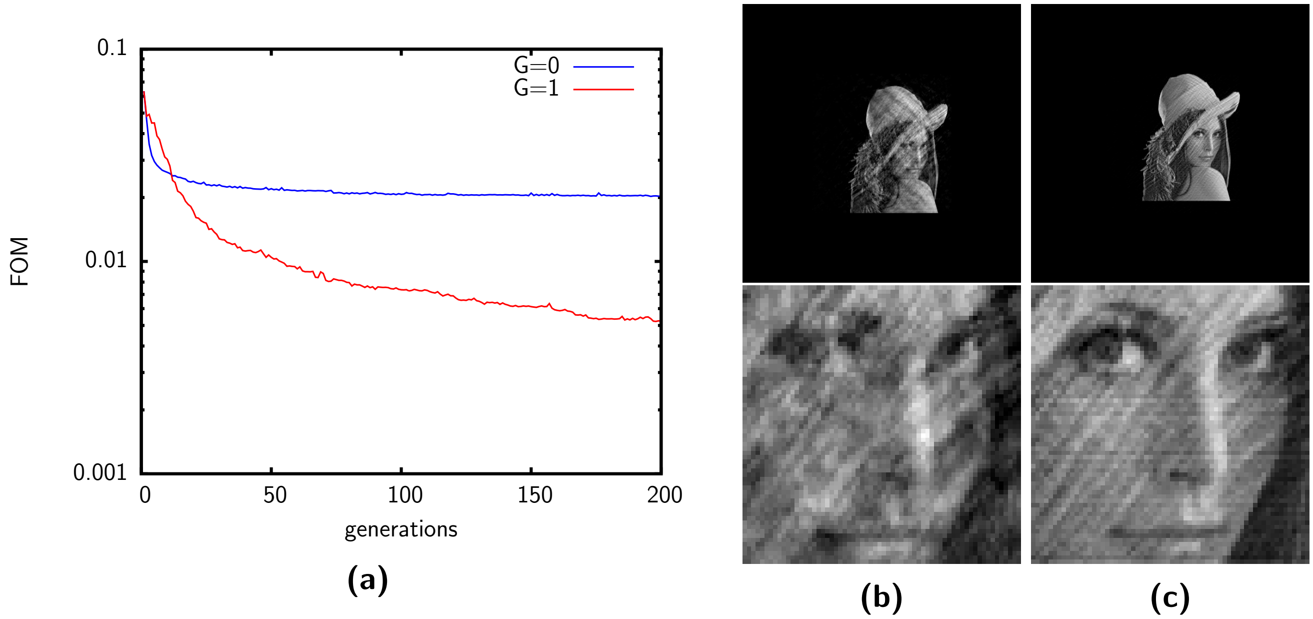

Figure S-2: Results of the real-valued test. (a) Retrieved function (above) and a zoom (below) for $G = 0$; (b) Retrieved function (above) and a zoom (below) for $G = 1$; (c) Figure of Merit.

Table S-1: MPR parameters related to Fig. S-2

Parameter	N_p	r	C	D_c	G	N_{HIO}	N_{ER}	R_c
Value	512	1.8	0.5	1	—	10	10	1

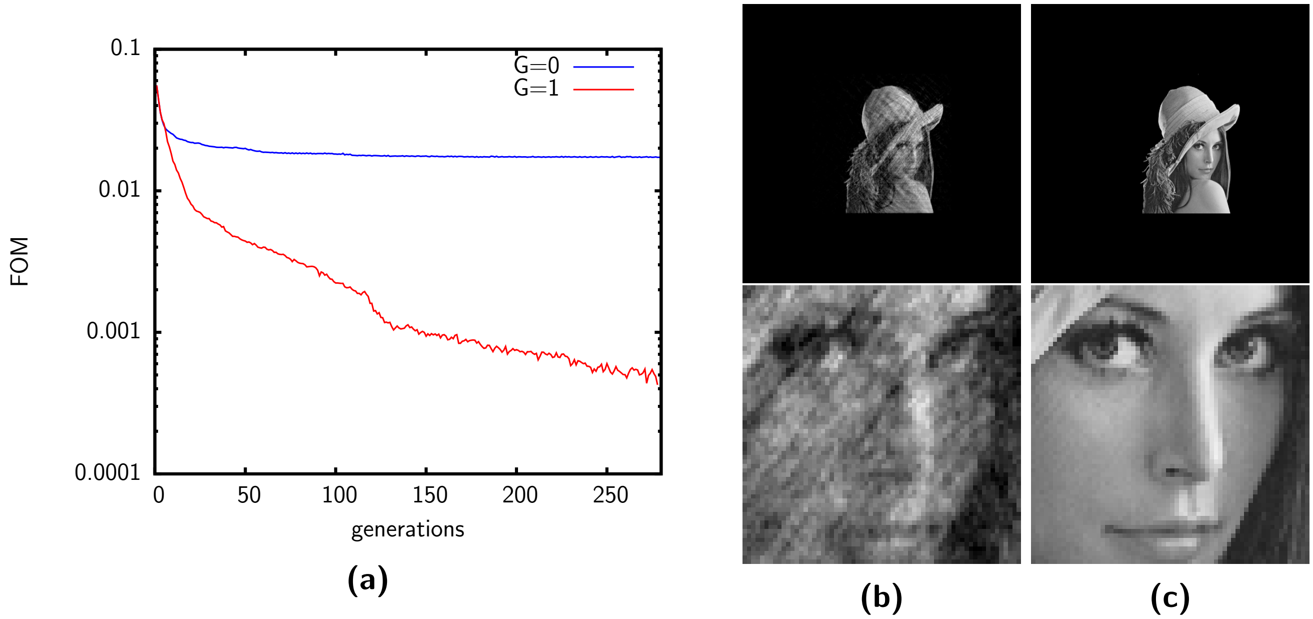

Figure S-3: Results of real-valued retrieval test. (a) Retrieved function (above) and a zoom (below) for $G = 0$; (b) Retrieved function (above) and a zoom (below) for $G = 1$; (c) Figure of Merit.

Table S-2: MPR parameters related to Fig. S-3

Parameter	N_p	r	C	D_c	G	N_{HIO}	N_{ER}	R_c
Value	16384	1.8	0.5	1	—	10	10	1

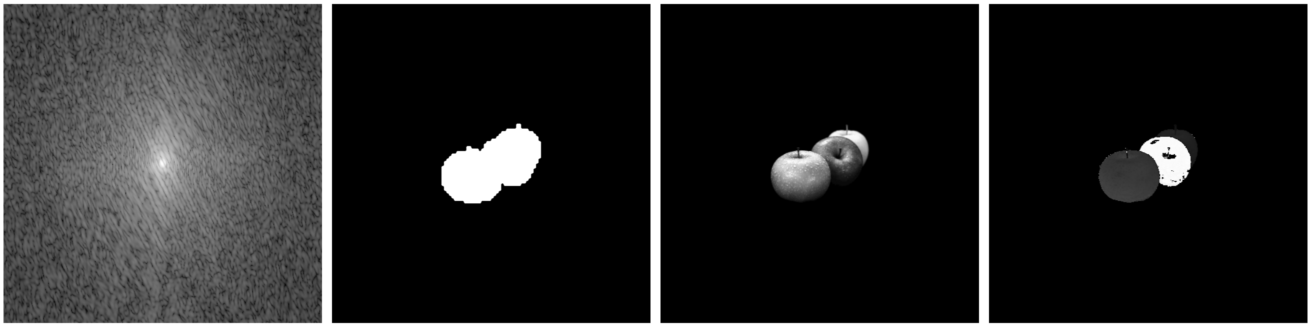

Figure S-4: Complex-valued retrieval: (a) Input diffraction pattern; (b) provided initial support function S; (c) true solution module ($|\rho(\mathbf{x})|$); (d) true solution phase ($\arg \rho(\mathbf{x})$).

zoom on the panel below; Fig. S-3a is the output of the FOM. Table S-2 reports the parameters used in this test of MPR.

The performance of the standard deterministic approach, i.e. $G = 0$, only slightly improves with respect to the situation depicted in Fig. S-2 where $N_p = 512$. Instead, the performance of MPR ($G = 1$) substantially improves with respect to the one depicted in Fig. S-2, providing a best trial solution very near to the true 2D function, starting by the square modulus of its FT, without using during the phase retrieval any information regarding the true support S of the object, which is remained always very loose, equal to the square area S_{max} depicted in Fig. S-1b.

For the population $N_p = 16384$, the *true error* $E_t[\rho]$ (see Eq. (S-3)) for $G = 0$ is 0.242, while for $G = 1$ is 0.007.

Phase Retrieval of a complex-valued function with a partial information about the support

The second test concerns a reconstruction where the solution $\rho_s(\mathbf{x})$ is complex-valued, i.e. the constraints described in Eq. (S-4) and Eq. (S-5) are not imposed. Data is, again, of size 512x512 pixels and have been assumed unaffected by any noise and any lack of information (adapted from the image at `http://openwalls.com/image?id=17210`, Copyright Creative Commons Attribution 3.0 Unported).

As depicted in Fig. S-4, we stored, in the real space representation of the solution, different information in the module, i.e. $|\rho_s(\mathbf{x})|$, and in the phase, i.e. $\arg[\rho_s(\mathbf{x})]$, in order to get a better visual comparison of the performances of the two different phasing approaches, standard ($G = 0$) and MPR ($G > 0$).

In particular Fig. S-4a shows the input diffraction pattern, Fig. S-4b the provided support function S, S-4c the true solution module ($|\rho(\mathbf{x})|$) and S-4d the true solution phase ($\arg \rho(\mathbf{x})$). As in the previous test, the input diffraction pattern is given by the square modulus of the FT of the 2D complex function to be retrieved.

As discussed in the main text, we applied MPR to an experimental case of Electron Diffractive

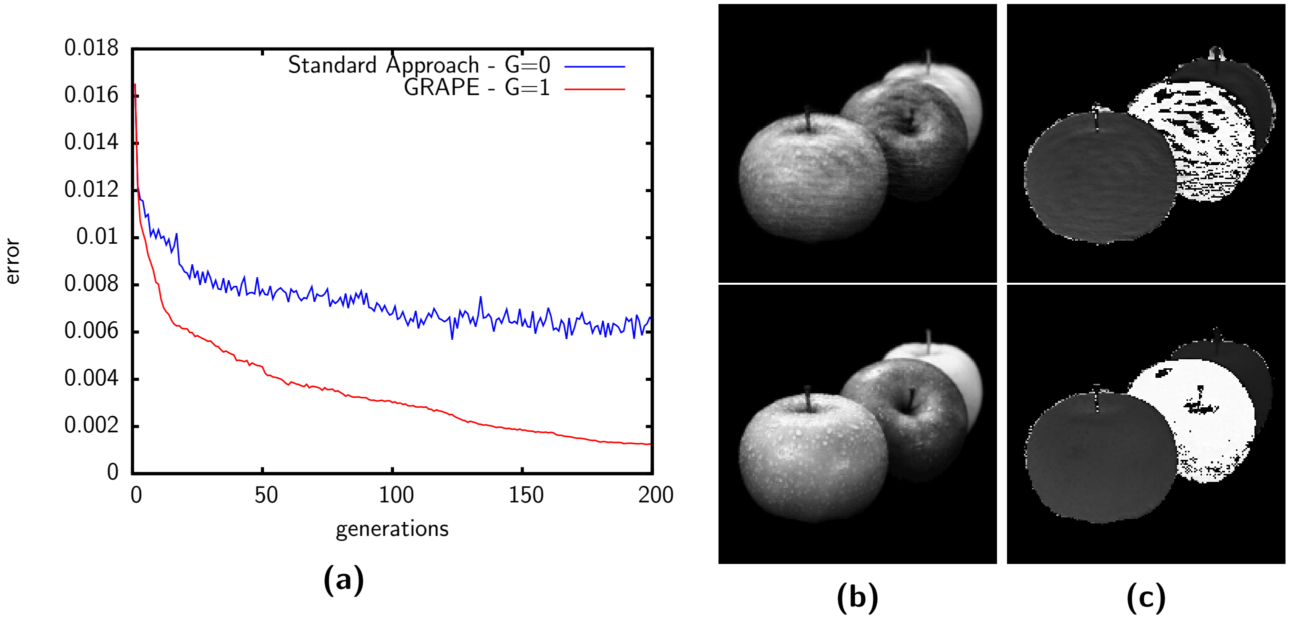

Figure S-5: Results of complex-valued retrieval test. (a) Figure of Merit; (b) Retrieved modulus in the real space, $|\rho_s(\mathbf{x})|$, of the best trial for $G = 0$ (above) and $G = 1$ (below); (c) retrieved phase in the real space, $\arg[\rho_s(\mathbf{x})]$, of the best trial for $G = 0$ (above) and $G = 1$ (below).

Table S-3: MPR parameters related to Fig. S-5

Parameter	N_p	r	C	D_c	G	N_{HIO}	N_{ER}	R_c
Value	1024	1.8	0.5	1	—	40	20	1

Imaging (EDI) [S.1]. Since the approximate knowledge of the support at a worse spatial resolution is a typical situation encountered in EDI, where S is obtained by the High Resolution Transmission Electron Microscopy (HRTEM) image, the support function has been extracted by thresholding the true solution (Fig. S-4c) after a resizing to 128x128 pixels. The area defined by the support function is 5%, which implies a *constraint ratio* [S.4] $\Omega = 10$.

Results of phasing are shown in Fig. S-5. Fig. S-5b shows the retrieved modulus in the real space, $|\rho_s(\mathbf{x})|$, of the best trial for $G = 0$ (above) and $G = 1$ (below); Fig. S-5c shows retrieved phase in the real space, $\arg \rho(\mathbf{x})$, of the best trial for $G = 0$ (above) and $G = 1$ (below); Fig. S-5a shows the Figure of Merit. Table S-3 reports the parameters used in this test of MPR which are the same both for $G = 0$ and $G = 1$ to compare the efficiency of the stochastic approach with respect to the deterministic one in the same conditions.

As already observed in the case of a real-valued 2D function phase retrieval, MPR outperforms the standard approach. It is useful to underline that the better performance of MPR is reflected not only in a better FOM, but appears evident in the whole reconstruction quality, that is the module of $\rho_s(\mathbf{x})$, $|\rho_s(\mathbf{x})|$, and the phase $\arg[\rho_s(\mathbf{x})]$ of the best trial solution.

Algorithm tuning

The tuning step, i.e. the regulation of the parameters for the reconstruction, is the most delicate step, because it is strictly dependent on the features of the experimental data and on the kind of solution we are trying to find. A phasing process can be, in general, divided into two main parts:

Table S-4: Summary of the different parameters present in MPR. Values are only indicative and the user is free to experiment any combination of them.

MPR parameters				
Parameter	Description	Typical values	Typical values for *ab initio* retrieval	Typical values for *refining* retrieval
N_p	Dimension of the *population*	Higher is better	—	—
r	Exponent of the *rigged roulette*	1.5 - 3	1.5 - 2.5	2 - 3
C	Balancing coefficient for the crossover	0.2 - 0.8	0.4 - 0.6	0.2 - 0.4
D_c	Differential coefficient for the crossover	0.5 - 1.5	0.8 - 1.5	0.5 - 1
G	Genetic fraction	0 - 1	0.3 - 1	0.6 - 1
N_{HIO}	Iterations of Hybrid Input-Output algorithm	0 - 20	5 - 20	0 - 5
N_{ER}	Iterations of Error Reduction algorithm	0 - 20	0 - 10	10 - 20
R_c	Coefficient for the initial setup	0.1 - 1	0.6 - 1	0.1 - 0.4

The first one can be called *ab initio* phase retrieval, where we don't have any good estimation of the solution: the aim of the algorithm is to explore widely the space and find a density which can be considered an acceptable estimation of the solution.

The second part can be called *refining* phase retrieval, where an acceptable estimation of the solution is present: in this case, the aim of the algorithm is to optimize the error functional around this good initial guess, with a dense exploration of the near space.

For these reasons, parameters must be set accordingly to the aim of the retrieval process, paying attention to the type of research that is being carried out, i.e., *ab initio* or *refining.* Table S-4 shows an overview on the parameters, giving an idea of their values also according to the scope of the retrieval process.

Typical retrieval process on EDI experimental data

In a typical retrieval process, the algorithm needs, as input, a diffraction pattern, a support function that defines the area of the real space where the reconstruction can have values different from zero and, if available, an initial guess (see the main text).

In the case of the $SrTiO_3$ reconstruction, described in the main text, the retrieval is *ab initio* at all: every element of the population is created from totally random phases as indicated by the value of the R_c coefficient (see Table S-5).

The behaviour of MPR is well depicted in Fig. S-6, which represents the Figure of Merit for the $SrTiO_3$ data. Due to the totally random initial guesses, the error of the reconstructions in the first generations is very sparse. With the progress of the iterations, differences between the various trial elements in $\{\rho_i(\mathbf{x})\}_{i=1\dots N_p}$ decrease and all the population converges to a specific error value. The number of generations needed to reach convergence can fluctuate a lot, depending on the parameters and on the data. Parameters favoring a wider exploration of the space cause a longer convergence time, while setting parameters to get a solution in a lower time can induce an undesired local stagnation of the algorithm.

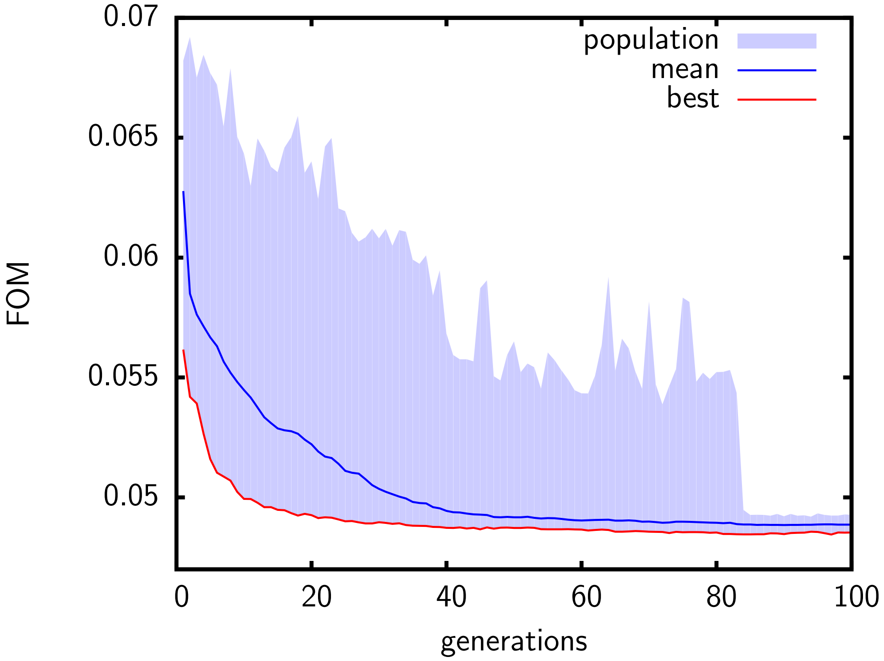

Figure S-6: Typical Figure of Merit of a MPR retrieving process: the light–blue area fills the space between the FOM of the best and the FOM of the worst trial.

Table S-5: MPR parameters related to $SrTiO_3$ reconstruction described in the main text

Parameter	N_p	r	C	D_c	G	N_{HIO}	N_{ER}	R_c
Value	1024	1.8	0.4	1	1	4	16	1

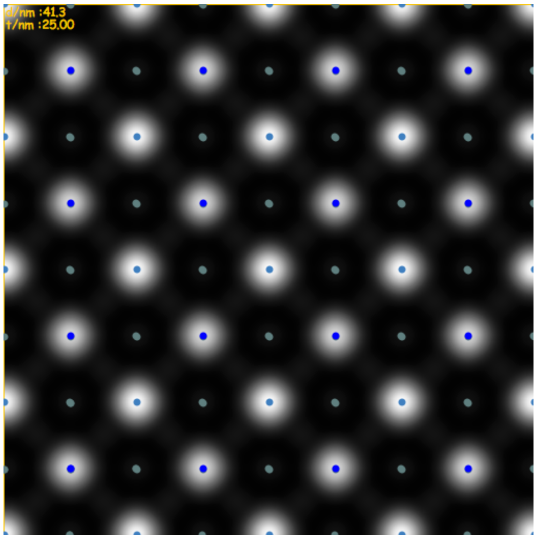

Figure S-7: HRTEM image simulation of $SrTiO_3$ in [100] zone axis for underfocus 41.3 and specimen thickness of 25nm, corresponding to the experimental image shown in the main text. The dots in the figure point out the structural positions of the atomic species in this projection: Sr=Blue, Ti+O=pale blue, O=pale green. It should be noted that for these experimental conditions the oxygen atomic columns are not visible.

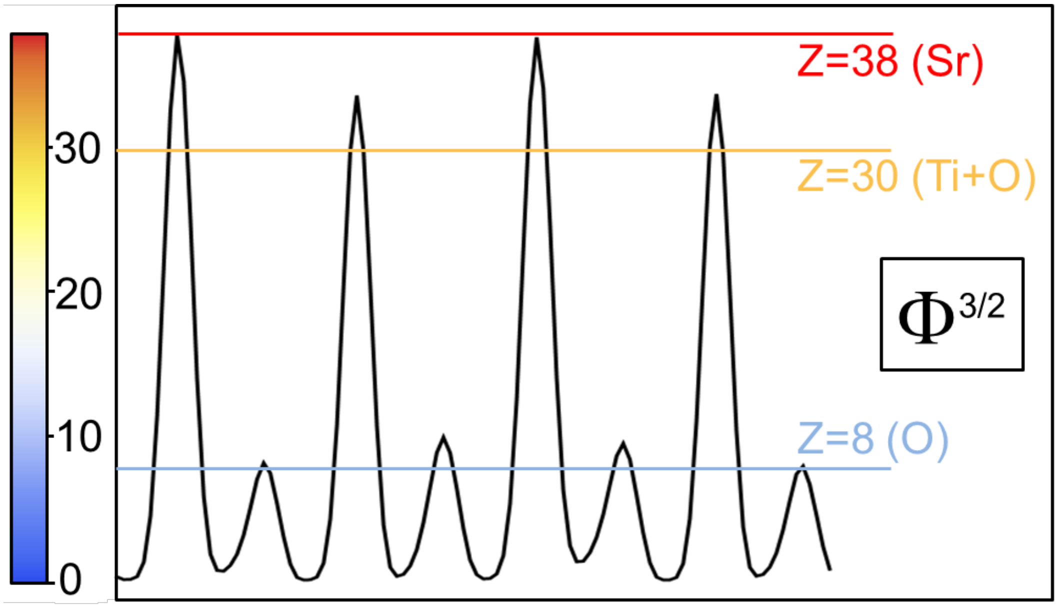

Figure S-8: A scan of $\Phi^{\frac{3}{2}}$, obtained by Fig.8d, normalized to have the maximum value equal to 38.

Further considerations on KEDI results

Let us observe that the projected atomic potential Φ is approximately proportional to $Z^{\frac{2}{3}}$, with Z the atomic number [S.5].

In Fig.S-8 we have shown a scan of $\Phi^{\frac{3}{2}}$, obtained by Fig.8d, normalized to have the maximum value equal to 38, i.e. to the atomic number of the heaviest atoms of the considered structure. From Fig.S-8 it can be evinced the accuracy with which the KEDI projected atomic potential is retrieved by the MPR approach. The spatial resolution scale is sub-atomic (less than 0.1 nm) [S.2]. The so normalized $\Phi^{\frac{3}{2}}$ map, in the central region of the nano-beam, gives maximum values ranging between 8 and 10 for oxygen atomic columns, with an average value close to 9.

Thus, the *accuracy* with which the less intense oxygen atomic columns can be retrieved, with respect to the true value of $38 \times 0.35^{\frac{3}{2}} = 7.9$ is $(9.1 - 7.9) \div 7.9 = 0.15$, i.e., with a systematic overestimation of the 15%.

Indeed, an oscillation in the brightness on the oxygen columns has to be expected as it depends on the number of atoms in the column and the presence of oxygen vacancy, well known in STO, would hence change the relevant intensity in the crystal potentials for the oxygen columns.

Furthermore it should be noted that the crystal potential calculations performed by linear combination of atomic potentials are affected by an error of about 10% [S.3]. Moreover, theoretical projected atomic potentials are only approximately proportional to the atomic number [S.5]. Finally, the *precision* with which the oxygen atomic potential can be locally retrieved is of ± 1 on an average value of about 9, i.e., slightly larger than 10%.

Moreover, our tests show that either an inaccurate determination of phases or a wrong support prevent the correct retrieval of the solution. This is true also for the determination of the atomic structure of the $SrTiO_3$ in the considered zone axis ([100]). Indeed, a wrong support leads to inaccurate phase retrievals which, in turn, leads to both incorrect relative intensities of columns belonging to different atomic species and to incorrect positions of the less intense ones, i.e. of the oxygen columns, with respect to Sr and Ti+O columns. The tests discussed in the Main

section have shown that the MPR approach proposed in this work is less dependent by the initial inaccuracy of the support knowledge, with respect to deterministic phase retrieval schemes. Indeed, our simulations have shown that using a genetic phase retrieval even by starting by a not well defined support the correct solution can be retrieved. This finding is very important to reach, via KEDI or EDI approaches, the maximum possible accuracy in the experimental determination at the sub-atomic scale of the structure of the material under investigation.

References

[S.1] Liberato De Caro, Elvio Carlino, Gianvito Caputo, Pantaleo Davide Cozzoli, and Cinzia Giannini. Electron diffractive imaging of oxygen atoms in nanocrystals at sub-angstrom resolution. *Nat Nano*, 5(5):360–365, May 2010.

[S.2] Liberato De Caro, Francesco Scattarella, and Elvio Carlino. Determination of the projected atomic potential by deconvolution of the auto-correlation function of tem electron nano-diffraction patterns. *Crystals*, 6(11):141, 2016.

[S.3] Earl J Kirkland. Advanced computing in electron microscopy. *J. Appl. Cryst*, 32:378–379, 1999.

[S.4] Rick P Millane and Romain D Arnal. Uniqueness of the macromolecular crystallographic phase problem. *Acta Crystallographica Section A: Foundations and Advances*, 71(6), 2015.

[S.5] Boris Konstantinovich Vainshtein. Structure analysis by electron diffraction. 2013.

[S.6] A. G. Weber. The usc-sipi image database version 5. *USC-SIPI Rep.*, 315:1–24, 1997.
